# Moderate Exercise Plus Sleep Education Improves Self-Reported Sleep Quality, Daytime Mood, and Vitality in Adults with Chronic Sleep Complaints: A Waiting List-Controlled Trial

**DOI:** 10.1155/2011/809312

**Published:** 2011-11-24

**Authors:** Carmen Gebhart, Daniel Erlacher, Michael Schredl

**Affiliations:** ^1^Institute of Sports and Sports Science, Heidelberg University, Im Neuenheimer Feld 700, 69120 Heidelberg, Germany; ^2^Institute for Sport Science, University of Bern, Bremgartenstrasse 145, 3012 Bern, Switzerland; ^3^Sleep Laboratory, Central Institute of Mental Health, J5, 68159 Mannheim, Germany

## Abstract

Research indicates that physical exercise can contribute to better sleep quality. This study investigates the six-week influence of a combined intervention on self-rated sleep quality, daytime mood, and quality of life. A nonclinical sample of 114 adults with chronic initiating and the maintaining of sleep complaints participated in the study. The intervention group of 70 adults underwent moderate physical exercise, conducted weekly, plus sleep education sessions. Improvements among participants assigned to the intervention group relative to the waiting-list control group (*n* = 44) were noted for subjective sleep quality, daytime mood, depressive symptoms and vitality. Derived from PSQI subscores, the intervention group reported increased sleep duration, shortened sleep latency, fewer awakenings after sleep onset, and overall better sleep efficiency compared to controls. The attained scores were well sustained and enhanced over a time that lasted through to the follow-up 18 weeks later. These findings have implications in treatment programs concerning healthy lifestyle approaches for adults with chronic sleep complaints.

## 1. Introduction

Nocturnal sleeplessness in terms of prolonged initiation of sleep times, frequent nightly awakenings, early morning awakenings, or sleep that are chronically nonrestorative and the consequences on daytime functioning is referred to as insomnia (ICSD-2, 2005). In a review of 21 representative studies by Ohayon [1], the prevalence of insomnia symptoms ranges from 10% to 48% in the general population across the world, and 9% to 15% report additional daytime consequences (e.g., depressive mood and irritability). Insomnia, therefore, can be seen as being a notable public health problem. Disorders of the sleep-wake cycle not only have an acutely negative impact on daytime functioning but also, in the long run, heighten the risk of overall health-related quality-of-life impairment [2–4]. Chronic sleep problems are associated with serious health problems like psychological disorders, particularly depression [5, 6] and anxiety disorders [7] as well as medical consequences, for example, hypertension [8], diabetes [9], or obesity [10].

Insomnia has been treated either pharmacologically or with cognitive behavioural therapy. As a fact endorsed by the National Institute of Health [11], pharmacotherapy is effective for the acute management of chronic insomnia [12] although side effects must be taken into consideration, for example, dependence, tolerance, and daytime sedation [13]. Several meta-analyses of cognitive behavioural therapy have found similar short-term effects [14–16] but, most notably, reliable and mainly long-lasting sustained benefits in several components of sleep among all age groups in comparison to pharmacotherapy [17–19]. Unfortunately, this multicomponent treatment places a few disadvantages (cf. [20]). For example, CBTs are quite unknown and little used among physicians [21]. Further, trained cognitive behavioral therapists are rare [22]. To bridge the gap between the high prevalence of insomnia and the low accessibility of CBT, regular physical activity has been proposed by sleep experts as another option for a better night sleep [23, 24].

The notion of sleep-promoting effects due to exercise has been documented in epidemiological surveys. For example, in an investigation carried out by Urponen et al. [25] with 1190 middle-aged adults in Finland, 44% of the participants listed exercise as the most sleep-promoting activity. Dose-response patterns of physical exercise for a better night of sleep have been found. For example, Brand et al. [26, 27] compared the sleep log data of 258 athletes with 176 controls (mean age 17.2 years) and found that high level exercise was related to better sleep patterns including higher sleep quality, shortened sleep latency, and fewer awakenings during the night. A lack of habitual exercise is associated with more reported sleep complaints [28–30].

Randomised, controlled exercise-intervention studies in adults suffering from complaints having to do with initiating and maintaining sleep are rare but yielded promising results on subjective [31, 32] and objective (polysomnography and actigraphy) measures of sleep quality [33, 34]. For example, King et al. [31] investigated the effects of moderate-intensity endurance training over 16 weeks. The 20 older sedentary adults (50 to 76 years) of the exercise group reported better overall sleep quality after the intervention as assessed by the Pittsburgh Sleep Quality Index (PSQI) when compared to a control group. In the study of Reid et al. [32], participants (mean age 61.6 years) in the physical activity plus sleep hygiene group (*n* = 10) showed better results in subjective sleep quality (PSQI score) than the control group with only the sleep hygiene intervention (*n* = 7).

The present intervention study focused on the effects of physical exercise on subjective sleep quality in insomnia sufferers. Similar to the study by Reid et al. [32], the first aim of this study was to examine the efficacy of a combined program that included physical exercise and sleep education on subjective sleep quality in adults with a long history of sleep complaints. In contrast to the study by Reid, we controlled the treatment effects using a waiting-list group, and we included a large sample size. It was expected that the intervention would yield marked effects on subjective sleep in comparison to a waiting-list control group. The second aim of the study was to look for the “long-term” clinical effectiveness of the intervention. Thus far, exercise intervention studies have not implemented follow-up measures. In this investigation, we expect that the positive effects will be maintained as shown with other cognitive behaviour-based interventions. The third aim was to study any additional benefits of the combined intervention. As sleep problems affect daytime functioning, we also expected improvements in daytime mood and general quality of life.

## 2. Methods

### 2.1. Participants

**Figure 1:**
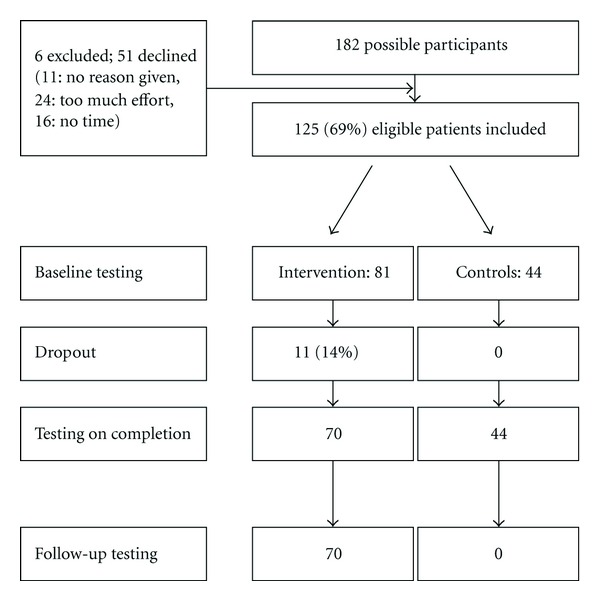
Flow chart of recruitment and completion of the study.

A total of 182 persons responded to the study recruitment. Six interested persons did not conform with the study criteria; eleven did not respond or could not be reached again after being given study information; for 16, the given time frame did not match with the offered time of the treatment; 9 persons did not want to participate after having received oral study information and 15 after receiving a written study invitation. Eleven participants did not finish the intervention (see Figure 1). A sample of 114 volunteers, 28 men and 86 women, (*M*_age_ = 56.0, SD_age_ = 11.7, age range: 17–77 years) were, thus, included for the purposes of the present study. Overall, *n* = 70 individuals completed the intervention and *n* = 44 joined the waiting-list control group. Participant characteristics requested by telephone screening are described in Table 1. No baseline differences were found.

### 2.2. Design

This study used a waiting-list-controlled design. In consideration of their availability, individuals were allotted either to the intervention group or a waiting-list control group. Measurements were collected at baseline, following intervention (after 6 weeks), and follow-up (3 months after following intervention). Participants of the waiting-list control group completed the same registrations at baseline and after a six-week waiting period without any intervention. At the end of the 6-week period, all control participants were offered the intervention. Because of this procedure, no follow-up measurement for the control group was possible.

### 2.3. Procedure

Participants volunteered to participate in this research by responding to advertisements in local print media. The initial screening of the research participants was conducted via telephone interviews. Eligibility criteria included the following: (1) difficulties in initiating sleep and/or maintaining sleep and/or early morning awakenings with difficulties returning to sleep for at least 3 months prior to study enrolment, (2) complaints of a nonrestorative sleep or negative impacts on daytime alertness, (3) free of any medical contraindications that would prevent regular, moderate Nordic walking, and (4) being able to speak and understand German sufficiently to provide informed content. No age restrictions were applied.

To ensure a nonclinical population, research participants were not excluded if suffering from either coexistent physical or psychological disorders nor when receiving chronic or hypnotic medication consumption. As cessation of hypnotic medication use is often difficult due to a rebound effect [13], participants were encouraged to continue their chronic intake as usual while participating in the study and under consultation with a physician.

All participants provided written informed consent. The study has been carried out at the Institute for Sport and Sports Science in Heidelberg and at the Central Institute of Mental Health in Mannheim.

### 2.4. Intervention

All participants received a combined 6-week intervention consisting of sleep education and physical exercise. The 6 weekly sessions were conducted in groups of 8 to 12 individuals. In total, each session lasted for 120 minutes whereas the first 60 minutes were about sleep education in a classroom and the second 60 minutes were an instructed moderate physical exercise (Nordic walking) outside. The sleep education and the Nordic walking sessions were provided by a sport scientist with a strong background in sleep research and psychology. Participants demonstrated good overall adherence (91.2%) to the weekly intervention program with a mean of 5.47 (SD 8) across the 6-week period.

#### 2.4.1. Sleep Education

The sleep education included an oral presentation (ca. 30 minutes) followed by a question-and-answer period (ca. 30 minutes). The sleep education information was based on guidelines for group programs for insomnia published by Riemann and Backhaus [35]. For example, the presentation included an overview about normal human sleep, normative changes in sleep-wake patterns occurring over the course of a life span, and also information about different sleep disorders, possible causes, and impacts on the quality of life. Participants also received written material about sleep education including, for example, a sleep hygiene checklist. They were encouraged to practice sleep hygiene instructions during the entire duration of the study. In later sessions, the participants also received information about further sleep-promoting behavioural (e.g., stimulus control) and cognitive methods (e.g., cognitive restructuring). Those techniques were demonstrated in class; however, it was optional in practice for the participants at home.

#### 2.4.2. Physical Exercise

The exercise program was based on current public health recommendations for adults [36]. Participants joined one weekly, instructed moderate aerobic session (Nordic walking) of 60 minutes duration. For the remaining two home-based exercise sessions per week, participants were instructed to engage in Nordic walking training or equivalent sports (endurance sports outside) of more than 30-minute duration and moderate impact. The subjective level of exertion was determined with the Borg Scale of Perceived Exertion [37]. Engagement in moderate exercise would aim for a Borg Scale level of “somewhat hard.” Beside the Borg Scale, the compliance of the physical exercise sessions at home was registered via an exercise log. Technique instructions and revisions were continuously provided by the project leader.

### 2.5. Measures

#### 2.5.1. Sleep Measures

The Pittsburgh Sleep Quality Index (PSQI) is a self-report instrument for assessing sleep disturbances and evaluates sleep quality retrospectively over a one-month period. Nineteen individual items are used to generate seven-component scores, each weighted equally on a 0 to 3 scale: subjective sleep quality, sleep latency, sleep duration, habitual sleep efficiency, sleep disturbances, use of sleeping medication, and daytime dysfunction. The sum of the scores for these seven subscales yields one global score of overall sleep quality and ranges from 0 to 21. According to Buysse et al. [38], a global score higher than 5 (cutoff) divides participants into poor and good sleepers, respectively, whereby greater scores indicate higher levels of sleep-related symptoms. The German adaptation was provided by the German Sleep Society (DGSM).

The German sleep questionnaire B (SF-B) from Görtelmeyer [39] comprises 28 items measuring composite scores of five factors: sleep quality, feeling of being refreshed in the morning, emotional balance in the evening, psychological exhaustion in the evening, and sleep-related somatic symptoms during sleep. The composite scores (averages) ranged from 1 to 5 (1 = never; 5 = very often) since most scales of the sleep questionnaire are constructed as five-point Likert's scales. Sleep latency was measured on a six-point scale (1 = less than 5 min; 2 = 5 to 10 min; 3 = 10 to 20 min; 4 = 20 to 30 min; 5 = 30 min to 1 h; 6 = more than 1 h). The estimates refer to the previous 2 weeks. For the study, only the dimensions “sleep quality” (11 Items) and “feeling of being refreshed in the morning” (7 Items) were analyzed.

#### 2.5.2. Daytime Mood

The German version [40] of the Symptom Checklist of Derogatis (SCL-90-R) is a 90-item self-report symptom inventory assessing psychological symptoms in nine distress dimensions: somatisation, obsessive compulsive, interpersonal sensitivity, depression, anxiety, hostility, phobic anxiety, paranoid ideation, and psychoticism. Those dimensions reflect various types of psychopathology over the last seven days. Each of the questions must be answered using a 5-point Likert's scale ranging from 0 = not at all to 4 = extremely. A Global Severity Index (GSI; General Symptomatic Index) represents the mean score of all 90 items and yields an indicator of the current level of psychic distress. A value ≥1 in the GSI or in any specific subscale is suggestive of psychopathology (mild = 1.00–1.49; moderate = 1.50–1.99; severe ≥2.00). Beside the global score “GSI” for the study only the “depression” (13 items) and “anxiety” (10 items) dimensions were evaluated [41].

#### 2.5.3. Quality of Life

The Short-Form-36 Health Survey (SF-36) is a widely used and validated questionnaire to assess the health-related quality of life (HRQoL). The German version used in this study [42] is composed of 36 items that assess the following eight health domains within different scales: physical functioning, role limitations due to physical problems (role, physical), bodily pain, general health perceptions, vitality, social functioning, role limitations due to emotional problems (role, emotional), and mental health. The results of each component range from 0 to 100, with higher scores indicating better health.

### 2.6. Data Analysis

Data was analyzed for the *n* = 114 participants that completed the study. Statistical analysis was conducted using descriptive statistics, chi-square tests, and *t*-tests for independent means to determine intragroup differences at baseline. Difference scores were calculated for each outcome measure between baseline and post (intervention) and also between baseline and follow-up. Repeated measures (ANCOVA) were conducted to assess the group differences in baseline and post (intervention) scores. To control for gender and age, those variables were included as covariates. Statistical significance was defined as *P* < .05 using two-tailed tests. Effect sizes for *t*-tests were calculated following Cohen [43], with 0.49 ≥ *d* ≥ 0.20 indicating small (i.e., negligible practical importance), 0.79 ≥ *d* ≥ 0.50 indicating medium (i.e., moderate practical importance), and *d* ≥ 0.80 indicating large (i.e., crucial practical importance) effect sizes. Effect sizes for ANCOVAs (partial eta-squared: *η* ^2^) were calculated following Cohen [43, 44], with 0.059 ≥ *η* ^2^ ≥ 0.01 indicating small (i.e., negligible practical importance), 0.139 ≥ *η* ^2^ ≥ 0.06 indicating medium (i.e., moderate practical importance), and *η* ^2^ ≥ 0.14 indicating large (i.e., crucial practical importance) effect sizes (see also [26]).

## 3. Results

### 3.1. Sleep Quality

**Figure 2:**
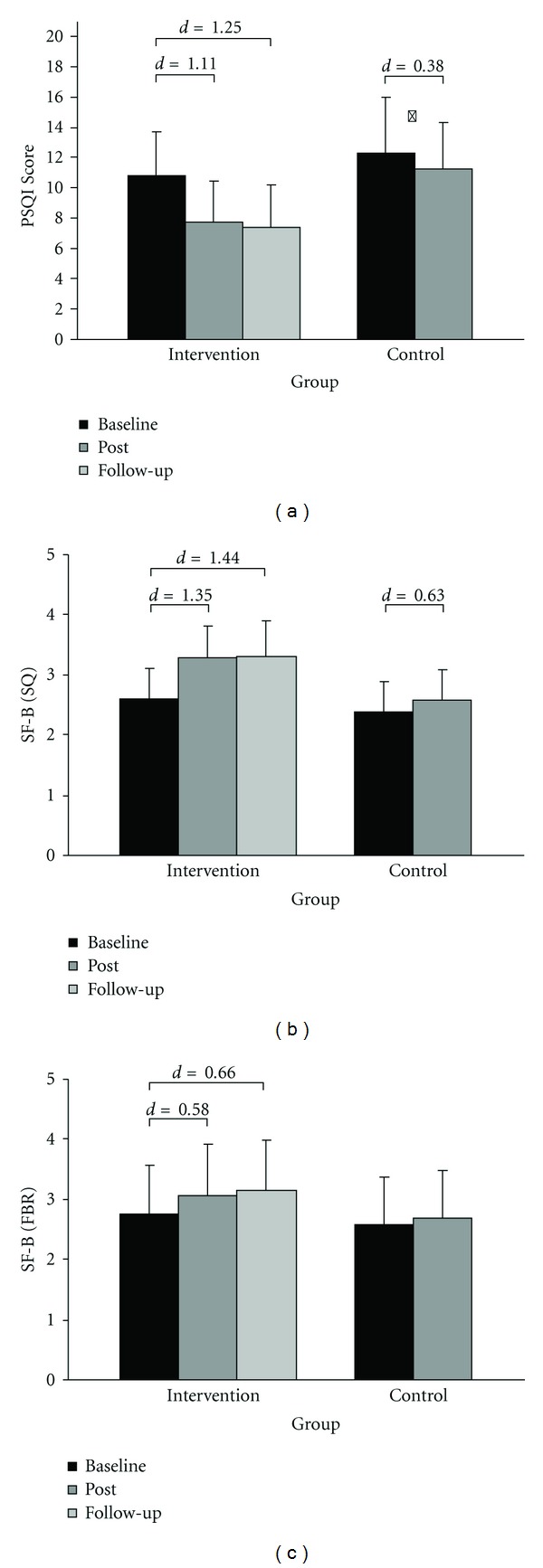
Mean and standard deviations for subjective sleep quality (SQ) and feeling of being refreshed in the morning (FBR) for the intervention and waiting-list control group. Lower rates indicate better sleep (PSQI). Effect sizes (Cohens *d*) are demonstrated for significant *t*-tests *P* < .001, *P = .015.

The means and standard deviations for the sleep measures of the Pittsburgh Sleep Quality Index (PSQI) and sleep questionnaire B (SF-B), with the factor “sleep quality” (SQ) and “feeling of being refreshed in the morning” (FBR) at baseline, after intervention, and follow-up are depicted in Figure 2.

For the global score of the PSQI (see Figure 2(a)), sleep quality of the intervention group improved from baseline to after intervention, *t*(69) = 8.91, *P* < .001, *d* = 0.94, and from baseline to follow-up assessment, *t*(67) = 10.61, *P* < .001, *d* = 1.25. A better global score of the PSQI was also found for the waiting-list control group, *t*(43) = 2.53, *P* = .02, *d* = 0.38; however, the improvement for the intervention group was more pronounced than that for the controls with an effect for Time by Group interaction, *F*(1,106) = 12.30, *P* = .001, *η* ^2^ = .10.

For SF-B (see Figures 2(b) and 2(c)), improvements among participants assigned to the intervention group were noted for SQ, *t*(60) = −10.49, *P* < .001, *d* = 1.35, and, for the factor FBR, *t*(61) = −4.54, *P* < .001, *d* = 0.58, after the intervention. At follow-up, intervention effects are still increased for SQ, *t*(59) = −11.18, *P* < .001, *d* = 1.44, as well as FBR, *t*(60) = −5.12, *P* < .001, *d* = 0.66, compared to baseline. SQ of the controls was also rated better, *t*(37) = −3.87, *P* < .001, *d* = 0.63, after the 6-week waiting period, but not FBR, *t*(37) = −1.67, *P* = .10. Again, the improvement was more pronounced for the intervention group than that for the waiting-list control group with a time by group interaction for SQ, *F*(1,94) = 18.96, *P* < .001, *η* ^2^ = .17, and FBR, *F*(1,95) = 3.05, *P* = .04, *η* ^2^ = .03.

Table 2 provides an overview of the descriptive and inferential statistics for the subscale values of the PSQI at baseline and following intervention. The intervention group reported better sleep quality, *t*(69) = 8.91, *P* < .001, *d* = 1.06, decreased sleep latency, *t*(68) = 4.87, *P* < .001, *d* = 0.59, longer sleep duration, *t*(67) = 5.18, *P* < .001, *d* = 0.63, and reached better scores in sleep efficiency, *t*(67) = 6.96, *P* < .001, *d* = 0.84 after the intervention. With respect to the time by group interaction, the improvements for sleep quality, sleep duration, and habitual sleep efficiency were higher compared to the waiting-list control group (Table 2, right column). When sleep time variables were analyzed as continuous variables (rather than subscores), there was a significant effect for sleep duration and sleep latency (Table 2, last two rows).

### 3.2. Daytime Mood and Quality of Life

Improvements in terms of additional benefits of the intervention group compared to the waiting-list control group for the Symptom Checklist of Derogatis (SCL-90-R) and the Short-Form-36 Health Survey (SF-36) are depicted in Table 3.

Looking at SCL-90-R, the intervention group improved their daytime mood as well as their overall psychological condition (Global Severity Index, GSI) following intervention, *t*(68) = 4.32, *P* < .001, *d* = 0.53, and at follow-up, *t*(66) = 5.81, *P* < .001, *d* = 0.71 when compared to baseline. They also demonstrated decreased depressive symptoms after the intervention, *t*(69) = 4.22, *P* < .001, *d* = 0.50, and hold up their level to follow-up, *t*(67) = 4.31, *P* < .001, *d* = 0.52. The control waiting-list group showed no improvements in any of the two parameters. There was an effect for time by group interaction for GSI, *F*(1,106) = 7.90, *P* = .01, *η* ^2^ = .07, and for the subscale depression, *F*(1,108) = 5.80, *P* = .02, *η* ^2^ = .05, and anxiety, *F*(1,108) = 3.39, *P* = .04, *η* ^2^ = .03.

Looking for possible changes in health-related quality of life (SF-36), the intervention group reported greater feeling of vitality, *t*(69) = −4.53, *P* = .001, *d* = 0.54, after the intervention with even greater improvements at follow-up, *t*(67) = −4.71, *P* = .001, *d* = 0.57, compared to baseline. The intervention group reached higher scores in mental health at follow-up compared to baseline, *t*(67) = −2.36, *P* = .02, *d* = 0.29, while the waiting-list control group showed no changes. There was a time by group interaction for vitality, *F*(1,112) = 6.87, *P* = .01, *η* ^2^ = .06.

## 4. Discussion

The results of the present investigation showed that a combined treatment with regular, moderate physical exercise plus sleep education is an effective method for improving subjective sleep quality, daytime mood, and vitality in chronic sleep-impaired adults.

The effect sizes which are comparable to CBT interventions studies [45, 46] indicate that the changes in subjective sleep quality (PSQI score, SF-B) are clinically relevant. Improvements achieved at the end of the intervention were well maintained over time and even enhanced three months later. These results are particularly encouraging given the chronic nature of the sleep complaints present in the study group and the relatively short intervention period. Participants of the intervention group needed up to 18 minutes less time to fall asleep, slept 33 minutes longer (47 minutes at follow-up), and reported an overall improvement in sleep efficiency of 13.4%—early to norm values [47]—after finishing the intervention. The current study achieved an average 3.1 point reduction in the global PSQI score which is comparable to the findings of King et al. [31] with an average reduction of 3.3 after an 16-week moderate endurance exercise intervention with 4 weekly sessions of >30 minutes duration. Reid et al. [32] demonstrated a slightly stronger effect of a 4.8 point reduction. This difference might be explained by the more restrictive exclusion criteria used in the Reid study; for example, participants were included when they showed a habitual sleep duration of less than 6.5 hours a night, which was verified with wrist actigraphy and a sleep log.

Comparing the calculated effect sizes of our study to meta-analytic findings of behavioural intervention studies indicates slightly lower scores for sleep onset latency, higher effect sizes for frequency of nocturnal awakenings, and similar effects in subjective sleep quality and total sleep time [15, 48]. Furthermore, the reduction in the use of hypnotics for the intervention group in the present study was consistent with previous cognitive behavioural therapy studies [49]. This clearly indicates that the intervention of our study is as effective as other approaches.

Unfortunately, to our knowledge, no systematic data exist from nonpharmacological intervention studies for parameters like feeling of being refreshed in the morning (FBR), which can be conceptualized as an indicator for restorative sleep—one of the most important diagnostic criteria for insomnia (ICSD-2, 2005). Therefore, the considerable improvement of the intervention group with regard to FBR is an important finding of the present study. Along the same line, Gerber et al. [50] found in a sample of young adults that participants with high fitness levels and no perceived lack of physical activity felt more restored after awakening and reported better mood in the morning. Moreover, Gerber et al. [50] were able to show that the relation between exercise and sleep seemed to be mediated via cognitive-emotional processes. Therefore, in future studies such measures should be included to explain the effects of physical exercise on sleep.

In this study, the most notable change in additional benefits was found for the global score of psychological distress (SCL-90-R) and mainly for the depression and anxiety dimensions. In comparison, Rybarczyk et al. [51] did not find any effects for depression and anxiety after a CBT intervention. However, a comparison with our study must be made cautiously because different measurement instruments for depression and anxiety were applied. In this regard, it must be considered that the data analyses in our study do not permit deciding why participants' sleep improved: it might be that exercise had a genuine impact on sleep. On the other hand, exercise might have had an impact on sleep as mediated via change in mood or dysfunctional thinking (e.g., Gerber et al. [50]). According to Harvey [52], the positive impact on daytime mood might be explained simply due to the experience of sleeping better. However, as Mota-Pereira et al. [53] showed, improvement of mood in dependence on moderate and regular exercise is far from being simply an “additional benefit.” To expect a straightforward answer about the effect of physical exercise and sleep quality, it must be considered that exercise also has a tremendous impact on physiological processes (see Deslandes et al. [54]; Puterman et al. [55]) and that the influence of exercise on both psychological and physiological processes is poorly understood so far (see also [56]).

In general, this study using a combined treatment program cannot distinguish the relative contributions of the physical exercise and sleep education components to the observed effects. Notably, in the study by Reid et al. [32], the sleep hygiene education which was provided for the control group did not show any effect on the sleep quality variables. Unfortunately, the duration of the sleep hygiene education in the Reid et al. [32] study is not clearly described. It seems that in our study the sleep education was longer and more detailed, and; therefore, it might be expected that this part of the intervention alone has already had positive effects on sleep (e.g., [57]). For example, as chronic sleep sufferers often feel helpless in managing their sleep problems, participating in the program may help them break the cycle of poor sleep hygiene, and the sleep education might have helped them to implement other healthy behaviours that could be conducive to sleep, such as avoidance of excessive alcohol consumption [58, 59]. Given the missing effects of sleep hygiene education on subjective sleep in the study by Reid et al. [32], the effects on sleep in the present study cannot be attributed to sleep education alone, but the physical exercise must have a prominent impact on sleep.

There are several hypotheses as to how exercise affects sleep, including the thermoregulatory, body restoration, and energy conservation hypotheses (cf. [59]). For example, the restorative theory predicts that an increase in energy expenditure will require a more intense sleep in order to recover, with more time spent in slow wave sleep or general longer sleep duration (e.g., [60]). In a recent work by Dattilo et al. [61], the authors hypothesized that sleep debt decreases the activity of protein synthesis pathways and increases the activity of degradation pathways. In other words, the catabolic process caused by exercise (e.g., damage to muscles) requires the restoration of muscle mass and muscle recovery. Muscle recovery would potentially be compromised because this process is strongly regulated by the previously discussed anabolic and catabolic hormones, which are strongly influenced by sleep. However, as mentioned before, given that the influence of exercise on both psychological and physiological processes is poorly understood, the direct impact of physical exercise on sleep might be far different (cf. [59]). For example, bright light exposure during outdoor activity might also have had a positive effect on the sleep-wake circadian rhythm of the participants [33].

Some of the effects of the present study might be explained by the findings indicating that regular physical exercise has numerous health enhancing [62] as well as psychological benefits [63]. As we did not exclude people from the study with comorbid medical or psychiatric conditions, improvements in daytime mood and, thus, sleep improvements might be induced through this indirect pathway. Additionally, the findings regarding the vitality dimension of the SF-36 with moderate effect sizes as well as the feeling of being refreshed in the morning (FBR) might reflect not only a better night of sleep [45] but also an improved physical fitness [64]. Similar, Brand et al. [26, 27] were able to show that, among a sample of adolescents, regular exercise was related to favourable sleep and favourable psychological functioning, such as increased curiosity and stress resistance, and lower depressive symptoms and perception of pain.

One of the limitations of the study is the fact that participants were not randomized as to their inclusion in one or the other of the two groups. The participants were assigned with respect to their time schedules in order to minimize drop-out rates. One might even assume that the treatment group consisted of persons who were eager to participate, that is, highly motivated, but this does not affect the validity of our findings because this would be the normal situation when implementing the program.

The participants of the waiting-list control group showed small improvements in sleep quality, however, much smaller than those of the intervention group. These improvements might be explained by the expectation that their sleep problems will be treated soon (cf. [65]).

In comparison to pharmacological interventions, the combined treatment may take marginally longer to become evident require more time regular practice, and it might even necessitate the motivation for an overall lifestyle change. However, it also has stable effects and no negative side effects. A nondrug approach like this treatment program is of special interest for elderly sleep sufferers in view of the additional risk factors, for example, changing physiology, medical conditions, polypharmacy, or night-time falls under sleep medication [66]. Overall the effects of regular physical exercise for successful aging are well established [67].

Taken together, the combined sleep training represents a potential alternative nonpharmacological treatment for younger to older adults (age range in the present study: 17–77 years) suffering from chronic problems in initiating and maintaining sleep. It would be interesting to expand the present findings to patients who were formally diagnosed with primary insomnia (ICSD-2). Further, as the present findings are based on subjective measures of sleep quality, the study design should complement with polysomnographic data.

The program combining sleep education and moderate exercise is suitable for use in various settings as, for example, adult education, and might be more appealing to persons with sleep complaints than formalized CBT programs. As persons who might have suffered from secondary insomnia due to medical conditions or mental disorders were also included in the present study and showed improvements, it would be worthwhile to also carry out controlled group exercise intervention trials in patients with different medical or psychiatric conditions and impaired sleep. Further investigations are needed to study the effects of the different intervention segments implemented in this program and learn more about the optimal amount, intensity, timing, and kind of physical exercise in order to provide detailed exercise recommendations for the participants. In view of the clear benefits, nonpharmacological interventions merit continued investigation among chronic sleep-impaired populations.

## 5. Implications for Practice

Everyone has at some point experienced getting insufficient sleep. Chronic sleep complaints over days, months, even years impair the overall quality of life. As the sleep-wake cycle is affected by multiple factors [68], diagnosing insomnia warrants very careful and complex evaluations [49, 69]. Among others, a good health status, active and healthy lifestyle seem to be one of the preventive factors for developing sleep problems and play an important role in improving insomnia symptoms [30, 70, 71]. Therefore, sleep education and physical exercise are not only applicable as a treatment—as shown in the present study—but also can be used for prevention and should be placed in health education and lifestyle change programs, respectively [72]. Physical exercise programs should be appropriate to one's body constitution and fitness level, especially when beginning, to strengthen ones; self-efficiency in order to overcome common barriers and maximize adherence.

## Figures and Tables

**Table 1:** Baseline characteristics of the study samples.

	Groups		Statistics	
Variables	Intervention group (*N* = 70)	Waiting-list control group (*N* = 44)		
	*M* (SD)	*M* (SD)	*t*	*P*

Age	55.3 (11.2)	57.2 (12.4)	0.87	.39
Duration of sleep problems (years)	11.9 (11.1)^a^	12.02 (9.6)^b^	0.05	.96

	*n* (%)	*n* (%)	*chi^2^*	*P*

Gender	18 men (25.7%)	10 men (22.7%)	0.13	.72
	52 women (74.3%)	34 women (77.3%)		

*Sleep problems*				
Solely problems with initiating sleep	5 (7.1%)	4 (9.1%)	0.14	.71
Solely problems with maintaining sleep	30 (42.9%)	25 (56.8%)	2.11	.15
Problems initiating and maintaining sleep	35 (50.0%)	15 (34.1%)	2.78	.10
Consumption of hypnotics	30 (42.9%)	22 (50.0%)	0.56	.46

*Comorbidities*				
Somatic (e.g., cardiovascular disease)	52 (74.3%)	38 (86.4%)	2.37	.12
Psychiatric (e.g., depression)	14 (20.0%)	6 (13.6%)	0.76	.38
Medication for comorbidities	49 (70.0%)	33 (75.0%)	0.34	.56

^a^ *n* = 45 respondents; ^b^ *n* = 33 respondents.

**Table 2:** Model estimated means (*M*), standard deviation (SD) and Time × Group effects for the PSQI subscales.

	Groups				Statistical analyses^1^					
	Intervention group		Control waiting-list group		Factor		Factor		Interaction	
	Baseline	Post	Baseline	Post	time		group		time × group	
PSQI subscale	*M* (SD)	*M* (SD)	*M* (SD)	*M* (SD)	*F*	*η* ^2^	*F*	*η* ^2^	*F*	*η* ^2^
Sleep quality	1.99 (0.53)	1.31 (0.58)	2.07 (0.59)	1.98 (0.46)	1.40	0.01	10.76***	0.09	18.25***	0.14
Sleep latency	1.71 (0.82)	1.30 (0.67)	1.93 (0.87)	1.82 (0.90)	0.28	0.00	4.09*	0.04	2.38	0.02
Sleep duration	1.46 (1.01)	0.94 (0.79)	1.77 (0.94)	1.66 (0.89)	0.61	0.01	2.89	0.03	7.04**	0.06
Habitual sleep efficiency	1.81 (0.97)	0.96 (0.97)	2.18 (1.06)	2.05 (0.96)	0.01	0.00	11.54***	0.10	11.33***	0.10
Sleep disturbances	1.40 (0.49)	1.27 (0.45)	1.61 (0.58)	1.45 (0.50)	0.00	0.00	1.72	0.02	0.00	0.00
Use of sleep medications	0.91 (1.20)	0.66 (1.09)	1.16 (1.29)	0.80 (1.21)	0.19	0.00	0.50	0.01	0.16	0.00
Daytime dysfunction	1.53 (0.70)	1.33 (0.63)	1.61 (0.75)	1.52 (0.73)	4.27*	0.04	2.04	0.02	1.50	0.01
Reported sleep duration (h)	5.71 (1.21)	6.26 (1.06)	5.44 (1.00)	5.53 (0.90)	0.15	0.00	1.65	0.02	6.07*	0.05
Reported SOL (min)	36.80 (41.78)	18.81 (15.75)	41.32 (52.93)	36.32 (47.52)	2.94	0.03	0.63	0.01	4.85*	0.04

^1^ANCOVA with the factors “time” (depicted) “group” (depicted) and “gender” (not depicted) and the covariate age (two-tailed).

*P < .05; **P < .01; ***P < .001.

**Table 3:** Model estimated means (*M*), standard deviation (SD) and Time × Condition effects for daytime mood, and quality of life.

	Conditions				Statistical analyses^1^					
	Intervention group		Control waiting-list group		Factor		Factor		Interaction	
	Baseline	Post	Baseline	Post	time		condition		time × condition	
	*M* (SD)	*M* (SD)	*M* (SD)	*M* (SD)	*F*	*η* ^2^	*F*	*η* ^2^	*F*	*η* ^2^
*SCL-90-R*										
Depression	1.22 (0.66)	0.93 (0.61)	1.10 (0.86)	1.05 (0.81)	2.68	0.02	0.01	0.00	5.80*	0.05
Anxiety	0.90 (0.63)	0.66 (0.62)	0.88 (0.73)	0.87 (0.74)	6.00*	0.05	0.41	0.00	3.39*	0.03
Daytime mood (GSI)	0.98 (0.47)	0.77 (0.45)	0.95 (0.60)	0.92 (0.62)	2.40	0.02	0.39	0.00	7.9**	0.07

*SF-36*										
Physical functioning	88.17 (15.16)	88.25 (15.67)	84.24 (20.36)	85.59 (15.29)	0.07	0.00	2.53	0.02	0.12	0.00
Role, physical	71.74 (34.01)	75.73 (37.12)	68.60 (39.37)	69.77 (34.74)	5.20*	0.05	2.66	0.02	1.39	0.01
Bodily pain	69.54 (23.25)	71.89 (28.11)	68.57 (27.91)	66.48 (25.13)	0.04	0.00	2.29	0.02	2.49	0.02
General health	61.92 (17.25)	64.88 (17.94)	56.86 (18.82)	56.47 (18.64)	2.07	0.02	4.81*	0.04	0.52	0.01
Vitality	47.01 (17.10)	54.02 (17.46)	44.77 (19.50)	45.76 (17.22)	0.00	0.00	2.88	0.03	6.18**	0.05
Social functioning	78.75 (21.00)	82.32 (18.97)	75.85 (23.72)	79.83 (23.38)	0.54	0.01	0.78	0.01	0.03	0.00
Role, emotional	74.51 (33.64)	79.41 (33.60)	65.53 (41.67)	71.21 (40.88)	0.03	0.00	2.91	0.03	0.06	0.00
Mental health	61.42 (15.28)	64.44 (14.64)	56.48 (17.26)	58.45 (18.83)	3.07	0.03	2.26	0.02	0.06	0.00

^1^ANCOVA with the factors “time” (depicted), “group” (depicted), and “gender” (not depicted) and the covariate age (two-tailed).

*P < .05; **P < .01; ***P < .001.
